# Enhancing the diversity of self-replicating structures using active self-adapting mechanisms

**DOI:** 10.3389/fgene.2022.958069

**Published:** 2022-07-26

**Authors:** Wenli Xu, Chunrong Wu, Qinglan Peng, Jia Lee, Yunni Xia, Shuji Kawasaki

**Affiliations:** ^1^ College of Computer Science, Chongqing University, Chongqing, China; ^2^ Chongqing Key Laboratory of Software Theory and Technology, Chongqing, China; ^3^ Faculty of Science and Engineering, Iwate University, Morioka, Japan

**Keywords:** self-replication, self-adaption, cellular automaton, gene mutation, biological resources

## Abstract

Numerous varieties of life forms have filled the earth throughout evolution. Evolution consists of two processes: self-replication and interaction with the physical environment and other living things around it. Initiated by von Neumann et al. studies on self-replication in cellular automata have attracted much attention, which aim to explore the logical mechanism underlying the replication of living things. In nature, competition is a common and spontaneous resource to drive self-replications, whereas most cellular-automaton-based models merely focus on some self-protection mechanisms that may deprive the rights of other artificial life (loops) to live. Especially, Huang et al. designed a self-adaptive, self-replicating model using a greedy selection mechanism, which can increase the ability of loops to survive through an occasionally abandoning part of their own structural information, for the sake of adapting to the restricted environment. Though this passive adaptation can improve diversity, it is always limited by the loop’s original structure and is unable to evolve or mutate new genes in a way that is consistent with the adaptive evolution of natural life. Furthermore, it is essential to implement more complex self-adaptive evolutionary mechanisms not at the cost of increasing the complexity of cellular automata. To this end, this article proposes new self-adaptive mechanisms, which can change the information of structural genes and actively adapt to the environment when the arm of a self-replicating loop encounters obstacles, thereby increasing the chance of replication. Meanwhile, our mechanisms can also actively add a proper orientation to the current construction arm for the sake of breaking through the deadlock situation. Our new mechanisms enable active self-adaptations in comparison with the passive mechanism in the work of Huang et al. which is achieved by including a few rules without increasing the number of cell states as compared to the latter. Experiments demonstrate that this active self-adaptability can bring more diversity than the previous mechanism, whereby it may facilitate the emergence of various levels in self-replicating structures.

## 1 Introduction

A cellular automaton (CA) is a discrete dynamical system that consists of a huge number of identical finite-state automata (Abou-Jaoudé et al., 2016; Xiao et al., 2020). Self-replication is a fundamental feature of life in biological resources, and it is a process of biosynthesis in which the original structure is replicated in the exact same structure (Cea et al., 2015; Baris et al., 2022; Gemble et al., 2022). Research of self-replication on CAs was founded by von Neumann (1966) and was viewed as one of the origins of artificial life research (Marchal, 1998; Gindin et al., 2014). In addition to reproducing offsprings with identical structures, attempts at including self-adapting mechanisms into the self-replicating models have been done (Suzuki and Ikegami, 2003; Sayama, 2004; Huang et al., 2013). In particular, Huang et al. (2013) designed a self-adaptive, self-replicating model using a greedy selection mechanism, which can increase the ability of the loops to survive through an occasionally abandoning part of their own structural information, for the sake of adapting to the restricted environment. Although the greedy mechanism is straightforward and sounds natural, it seems too passive. In addition to the self-adaptation which helps organisms survive (Williams and Burt, 1997), evolution and mutation are also inherent abilities of living things for adapting to environments in more active ways (Agrawal, 2001; Wilke et al., 2001; Miles et al., 2020; Moore et al., 2021; Monroe et al., 2022; Sasani et al., 2022), like the RNA virus (Domingo and Holland, 1997).

Likewise, identification of multiple adaptive mutations turns out to be essential for studying adaptation (Aminetzach et al., 2005; Scott, 2013; Lawson et al., 2020; Zuko et al., 2021). And, point mutations including insertions and replacements can help perform edits in human cells, thereby, in principle, correcting up to most of the known genetic variants associated with human diseases (Poduri et al., 2013; Anzalone et al., 2019; Buisson et al., 2019). Especially, changes in the self-replicating structure and behavior are controlled via their genetic memory (Bilotta and Pantano, 2006; Sha et al., 2020). As the living environment becomes more and more hostile, living organisms may have to change their own structures to survive. Self-adaptation through gene mutation, therefore, provides a spontaneous drive for natural life to survive against crucial competition with other living things and evolve into more advanced forms (Bilotta and Pantano, 2006; Sha et al., 2020). Moreover, self-adaptation has gained much attention in other fields such as knowledge architecture discovering (Edwards et al., 2009; Duan, 2019; Lei and Duan, 2021; Li et al., 2021) and edge computing (Xia et al., 2015; Song et al., 2018), due to its promise of more sophisticated and flexible computational paradigms (Duan et al., 2019a,b).

Inspired by the gene mutation-based self-adaptability in nature, this article endows two active mechanisms to the self-replicating loops which can facilitate the dynamical adaption of their structures to limited cellular regions. The new active mechanisms only need to change some rules in the passive model Huang et al. (2013), without increasing the number of cell states. The self-replication progress also contains two stages. In the first stage, the shape-encoding scheme is utilized to generate genetic information (construction signals), and the constructed arm receives the genetic codes to stretch forward, rightward, or leftward. During this period, collisions may occur at any moment and it seems urgently necessary to find a way out of a stalemate. Similar to the gene mutation process, we propose two solutions to resolve the collision. One mechanism generates, rather than waiting , a genetic code which resembles the insert mutation from single point mutation (Bargmann et al., 1986; Shenhav and Zeevi, 2020). Especially, the insertion of a transposable element can increase *Drosophila*’s resistance to an organophosphate pesticide (Aminetzach et al., 2005), which helps *Drosophila* to survive. In order to simplify the rules Huang et al. (2013), we randomly change the direction of the construction arms’ head. Another mechanism will choose to change following the genetic code from the mother loop next to the construction arm, which is similar to replace mutation (Vogel, 1972). The method of replacing genetic codes is used in suppression of tumorigenicity of human prostate carcinoma cells (Bookstein et al., 1990). After finishing the first extension stage of the construction arm, the mother loop will send a validation signal to the arm for the sake of confirming whether there is a closed loop or not. If it succeeds, the signal will cut off the link between the child loop and mother loop; otherwise, the construction arm will be drawn back. Finally, several typical and initial configurations are selected for the numerical experiments, which demonstrate that our new active mechanisms can obtain more types of variation loops, thereby increasing the opportunities of the organisms’ survival and expanding biodiversity (Klimentidis, 2012; Becerra-Rodríguez et al., 2021).

This article is organized as follows: Section 2 reviews related works. Section 3 gives an overview of the self-timed cellular automata and describes self-replicating loops with two active mechanisms which are capable of self-adapting their structures when the space is not enough to replicate themselves completely. Detailed comparison experiments are done in Section 4, followed by discussions given in Section 5.

## 2 Related works

Self-reproduction is one of the fundamental features in nature. Von Neumann was able to exhibit a universal Turing machine embedded in a cellular space using 29-states per cell and the 5-cell neighborhood. After that, many studies were done to reduce the complexity of the machine (Codd, 2014), re-mold signal-crossing organs (Buckley and Mukherjee, 2005), and realize self-replicating in the hardware (Merkle, 1992; Pesavento, 1995; Tempesti et al., 1998).

After ignoring the universality in computations, Langton (1984) proposed a simple self-replicating loop based on the periodic emitter (Codd, 2014) in a two-dimensional cellular space. Langton’s loop uses 8-states and 5-cell neighborhood (von Neumann neighborhood). After that, Langton’ loop attracts much attention and various attempts have been done, such as deleting the external sheath (Tempesti, 1995) or the inner sheath (Byl, 1989), producing unsheathed loops with less states (Reggia et al., 1993), and considering self-replication on asynchronous cellular automata (Nehaniv, 2002). Likewise, Ibáñez et al. (1995) introduced the ability of self-inspection, which allows the genome to dynamically construct concomitantly with its interpretation. Making full of the self-inspection ability, Morita and Imai (1996b) proposed a shape-encoding mechanism that depends on genetic codes from the loops’ phenotypical pattern to self-replication. Afterward, there were many studies in two-dimensional (Morita and Imai, 1996a) or three-dimensional reversible cellular space (Imai et al., 2002). In addition to self-replication, interacting between different loops has been conjectured, including self-protection with shielding, deflecting, and poisoning (Sayama, 2004), settling collisions with inroad, counter, defensive, and cancel methods (Suzuki and Ikegami, 2003). Such actions always harm the right of others to live.

All the aforementioned self-replicating models are based on synchronous CAs, in which all the cells are iterated to undergo state transitions simultaneously at every discrete time step. In nature, living systems are characterized by asynchronous timing modes, whereby studying self-replication on asynchronous cellular automata (ACAs) turns out to be crucial for a deeper understanding of the underlying mechanisms Huang et al. (2013). In an ACA, cells are updated at random timings independently from other cells, not needing a central clock signal to be distributed to all cells at any time. On the other hand, the unpredictable updating order of cells tends to bring more difficulty into the construction and self-reproduction on ACAs than on synchronous CAs. Nevertheless, Takada et al. (2007) designed a self-replicating loop based on the self-timed cellular automaton, which can self-reproduce parallelly and cope with the deadlock caused by collisions between self-replicating loops due to the asynchronous updating sequence. Especially, they used a simple mechanism that permits two colliding arms to fall back simultaneously. Huang et al. (2013) endowed a self-adaptive ability to the model, which allows two loops to not retract their arms but continue to accomplish self-replication when a collision occurs on occasion. In this case, the dead head will wait for a construction signal that can move the head into a direction away from the collision. More specifically, the choice of using which signal is made locally at the moment when the end of the constructing arm runs into an obstacle, and hence, such a selection is greedy. As a result, the passive self-adaptation can work in many situations where the normal reproduction of a loop is disturbed by some external constrain, thereby enabling the loop to survive and reproduce in a wide variety of regions (Huang et al., 2013).

## 3 Materials and methods

### 3.1 Self-timed cellular automata

**FIGURE 1:**
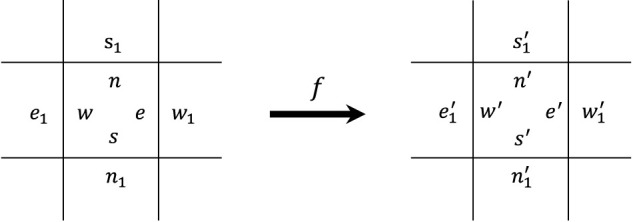
A transition rule according to the function *f*.

Our self-replicating loops are implemented on a self-timed cellular automaton (Peper et al., 2002; Takada et al., 2007), which comprises of a two-dimensional asynchronous cellular array of identical cells. Each cell is partitioned into four parts in a one-to-one correspondence with its neighboring cells, and each part has a state taken from a finite set of states at a time. Thus, a STCA may be deemed to a partitioned cellular automaton (Imai et al., 2002). Each cell undergoes transitions according to a transition function *f* that operates on the four parts of the cell and the nearest part of its four neighbors. The transition function *f* is defined as follows:

$$f\left(n,w,s,e,s_{1},e_{1},n_{1},w_{1}\right)=\left(n^{\prime },w^{\prime },s^{\prime },e^{\prime },s_{1}^{\prime },e_{1}^{\prime },n_{1}^{\prime },w_{1}^{\prime }\right),$$ (1)

where each value in parentheses denotes the new state of a partition after updating (see Figure 1).

Also, transition rules of an STCA are rotation symmetric, such that rotating both the left-hand side and the right-hand side of a rule in a multiple of 90° simultaneously give rise to equivalent rules of the original one. The transitions of cells in an STCA occur randomly and are independent of each other, i.e., an ACA. Because the update of a cell may change the nearest sub-cells of its neighboring cells, to prevent a write–conflict situation from occurring, we assume that all neighboring cells never undergo transitions at the same time. To this end, an effective scheme that can be used to iterate the STCA’s global transition is called random choice, by which at a time, only one cell is randomly selected with uniform probability to undergo a transition.

### 3.2 Self-replicating loops with active self-adaptability

Different from sheathed self-replicating loops in Suzuki and Ikegami (2003), a self-replicating loop implemented on our STCA model is unsheathed and needs the same number of states as the passive model in Huang et al. (2013). Four-cell states are used for each part of any cell, denoted by *♯*, ◦, ***•*** and ■, respectively. The state *♯* is often shown blank in the figures for convenience. A cell is quiescent if all of its four sub-cells are in the state *♯*. Transition rules are listed in Supplementary Appendix A, excluding the rotational symmetry equivalents.

#### 3.2.1 Normal self-replicating based on shape-encoding mechanism

When enough space is left, a loop can normally replicate itself in the cell region. Several signals listed in Table 1 are used to fulfill the self-replication according to the shape-encoding mechanism.

**TABLE 1:** The list of functions about various signals.

Name	Pattern	Function
Initiation signal	***•*** *♯*	Initiate self-replicating
Trace signal	*♯* ***•***	Trace the shape of a mother loop
Validation signal	***•*** ***•***	Validate whether the offspring and construction signals are replicated successfully
	◦ ◦	Advance construction arm straight forward
Construction signals	◦ ***•***	Advance construction arm leftward
	***•*** ◦	Advance construction arm rightward

**FIGURE 2:**
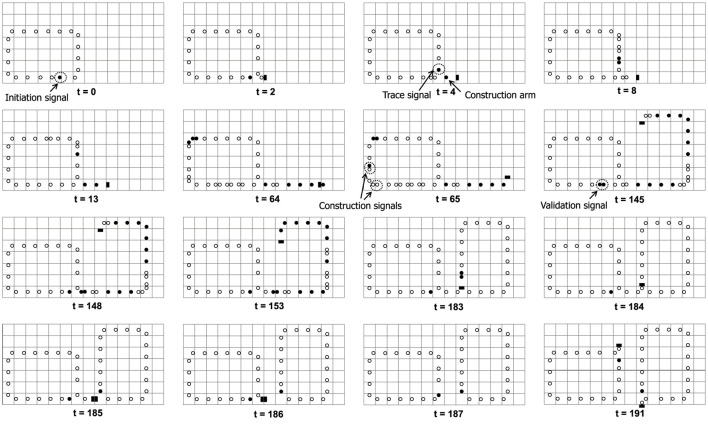
The normal process of self-replicating.

Figure 2 illustrates a typical self-replicating process of a loop, which is similar to Huang et al. (2013). An initiation signal will transmit counterclockwise before the replication starts. When the initiation signal arrives at a left-turn corner of the loop, it generates an initial construct arm stretching out from the corner, as well as an inspection head to trace the shape of the mother loop. The inspection head ***••*** will sequentially encodes each cell into an appropriate construction signals including going straight, turning right, and turning left. The signals from the mother loop are continuously transmitted to the head of the construct arm and are decoded into the corresponding part. Moreover, as soon as the shape-encoding process finishes, a validation signal is generated to verify whether the sub loop is constructed. If self-replicating succeeds, the signal will cut off the umbilical cord between the mother and the child, whereby both loops can start further replications individually.

### 3.2.2 Adaptive self-replication with mutations

**FIGURE 3:**
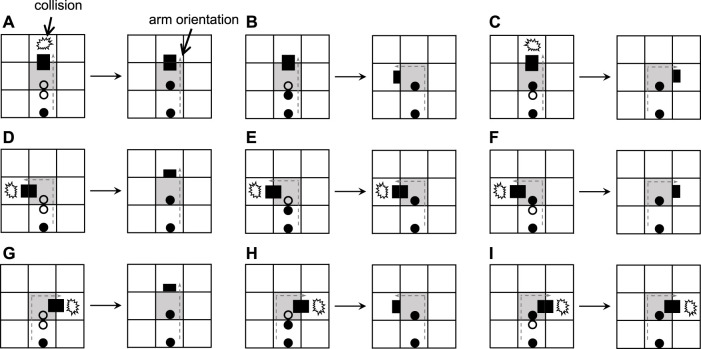
Transition rules of the greedy selection mechanism.

What will happen if there is no extra space for normal self-replication of a loop or if the space is taken up by the arms of other loops? Huang et al. (2013) considered a greedy selection mechanism to deal with the situation, which means only useful information is retained during self-replication. And the details are shown in Figure 3. After a collision occurs, the construction arm’s head becomes a dead head waiting for the construction signals coming from its mother. If the signal can work, then use it and change the direction of the construction arm. Otherwise, simply throw it away. Although such self-adaptation is simple and straightforward, it is passive and weak, resulting in much smaller child loops. In order to increase the adaptability and diversity of self-replicating models, we propose two novel mechanisms for active adaptation as follows:

Adding: add a different construction signal next to the head of the construction arm. For simplicity, the direction is directly changed at random.
Changing: change the construction signal following the head of the construction arm to other construction signals that are selected randomly.

Collisions are often inevitable due to the unpredictable nature of asynchronous updating. If the construction arm of a self-replicating loop perceives that the space is occupied, then it cannot extend furthermore and the state of the construction arm head will change from *♯*■ to ■■ (called dead end). There are many situations when a collision occurs, such as an arm bumping into another loop’s arm or an arm meeting the body of a loop.

**FIGURE 4:**
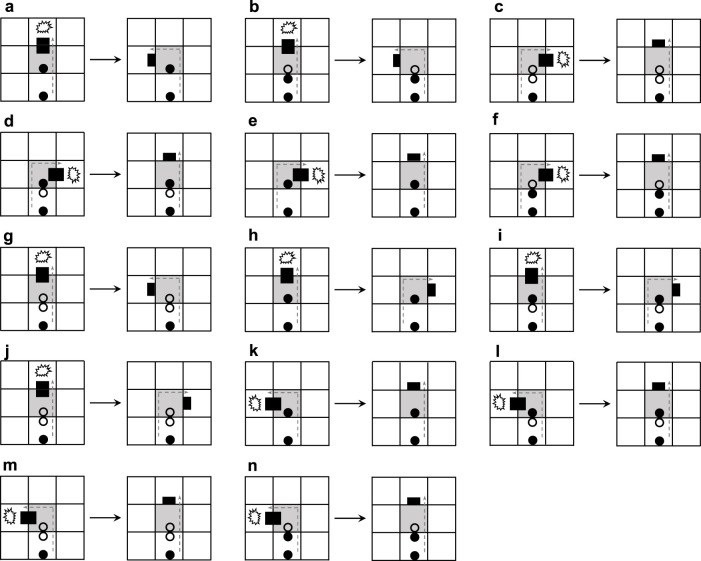
Transition rules of the adding mechanism.

Figure 4 elaborates the process of adding mechanisms for active adaptation. When the arm under going straight collides with an obstacle (Figures 4A,H), the current blocking state will be changed by randomly selecting one of the two orientations, namely turning left and turning right. Even a construction signal behind the dead head is a straight-going signal; the mechanism will add a random direction (Figure 4G and Figure 4J). Especially if the construction signal behind the dead head is a left-turning signal, the dead head will turn left and become normal after going straight is blocked (Figure 4B). Similarly, if there is a right-turning signal, the head will turn right (Figure 4I). Whatever a construction signal is behind the dead head, if the head is blocked by turning left or right, then the head will go straight.

**FIGURE 5:**
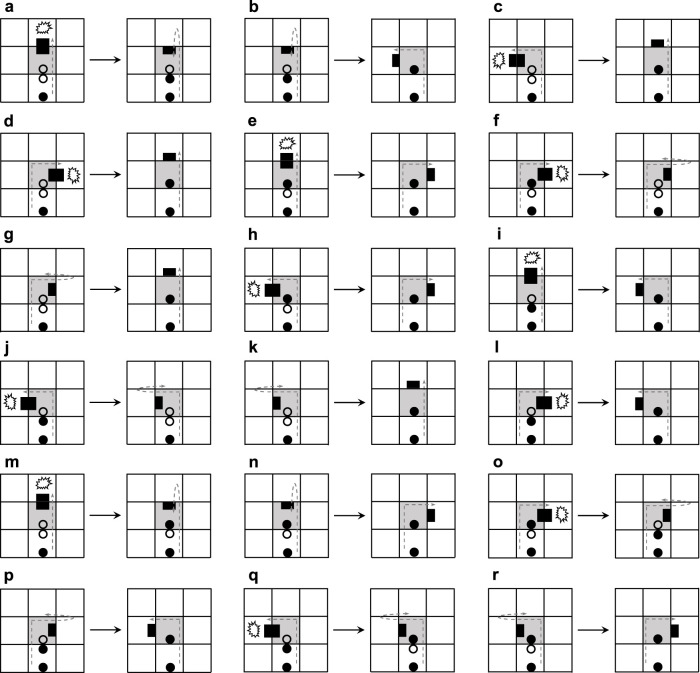
Transition rules of the changing mechanism.

The content of the changing mechanism is presented in Figure 5. If an arm going straight meets an obstacle and the construction signal behind the dead head is a straight-going signal, then the straight-going signal will change to a left-turning signal (Figure 5A) or a right-turning signal (Figure 5M) and the head goes back. Such a state is not durable, and after which the arm will turn left (Figure 5B) or turn right (Figure 5N). If the construction signal behind the dead head can mitigate the collision, the original signal remains constant (Figures 5C–E, H, and I). When the arm is blocked to turn left and the construction signal following the dead head is a left-turning signal, the construction signal will randomly mutate to a right-turning signal (Figure 5Q) or straight-going signal (Figure 5J). Similarly, the aforementioned situation also happens on turning right.

**FIGURE 6:**
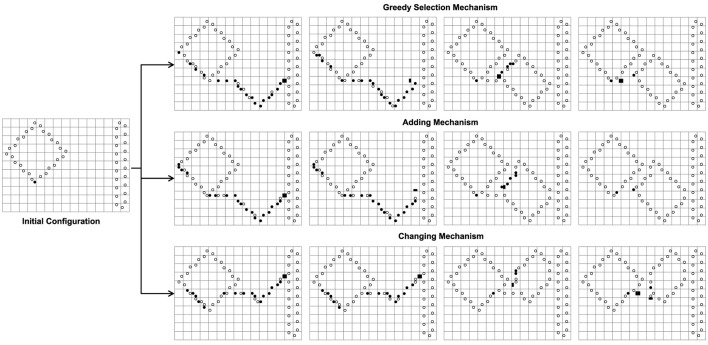
The different results of the greedy selection mechanism, adding mechanism, and changing mechanism starting from the same initial configuration where normal replication is limited by space.

We can see from Figure 6 that the greedy selection mechanism, adding mechanism, and changing mechanism can produce different sub-loops from the same initial configuration. Especially, the changing mechanism does not self-replicate at the beginning.

## 4 Experiments

In order to testify that active adaptation can produce more diversity of species than the previous passive adaptation, we set up various initial configurations and different boundary values to conduct the experiments. We used the trait distribution entropy from Sayama (2004) to characterize the diversity of the population, which shows as follows:

$$H=-\underset{i}{\sum }\left(\frac{n_{i}}{N}\log\frac{n_{i}}{N}\right)=logN-\frac{1}{N}\underset{i}{\sum }\left(n_{i}\;*\;logn_{i}\right),$$ (2)

where *n*_*i*_ is a quantity of loops that are made of *i* cells and *N* the number of loops in the current space. Moreover, the value of the trait distribution entropy ranges from 0 to *logN* and log function takes the logarithm base 10 instead of base *e*. *H* = 0 means that the space is filled with the same loop and *H* = *logN* can be obtained when each loop in the current space differs from each other (i.e., the value of each *n*_*i*_ is 0 or 1 for all *i*). Especially, loops which posses different manifestations belong to different species even if the loops consist of the identical number of cells.

**FIGURE 7:**
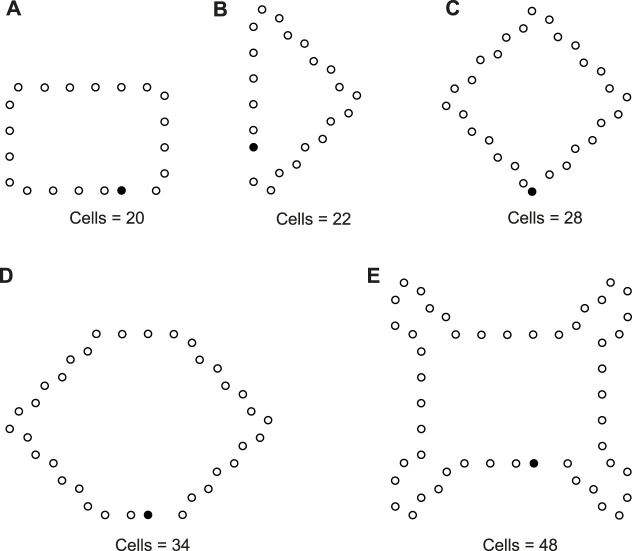
Different initial configurations.

**FIGURE 8:**
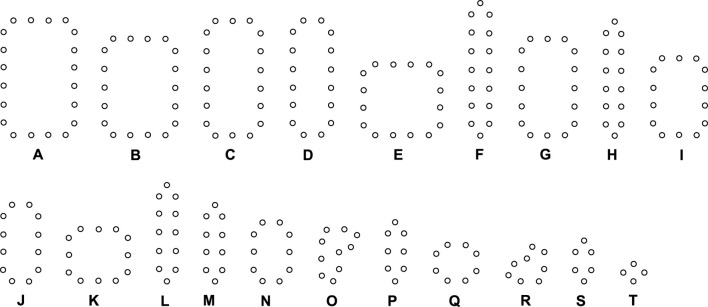
All final loop structures starting from the configuration in Figure 7A by the greedy selection, adding, and changing mechanisms.

We use different initial configurations to do experiments as shown in Figure 7, in which, the first three are common shapes and the last two are irregular. For simplicity, all possible final structures of replicated sub-loops starting from the initial configuration in Figure 7A by either self-adaptation mechanism are listed in Figure 8. In addition, the quantities and distributions of each structure in the cellular spaces using greedy selection mechanism, adding mechanism, and changing mechanism are provided in Tables 2, 3, and 4, respectively. As a result, compared with the other two active mechanisms, the greedy selection (passive) mechanism has a highest value of H in 80*65 cellular space, because the space is not filled with one or two identical and abundant small loops. However, on the whole, the adding mechanism and changing mechanism have higher values of H than the greedy selection mechanism.

**TABLE 2:** Statistical numbers of the loops with various structures for the greedy selection mechanism on different cellular spaces starting from the initial configuration in Figure 7A.

Loop Size	Shape∖Amount∖Space	60*60	80*65	100*65	Loop Size	Shape∖Amount∖Space	60*60	80*65	100*65
20 cells	Figure 8A	55	63	104	10 cells	Figure 8M	1	42	1
16 cells	Figure 8D	0	1	1		Figure 8N	2	4	20
	Figure 8E	13	7	2	8 cells	Figure 8P	4	0	3
14 cells	Figure 8H	0	13	0		Figure 8Q	3	5	11
	Figure 8I	6	6	3	6 cells	Figure 8S	2	33	16
12 cells	Figure 8J	9	6	9	4 cells	Figure 8T	2	19	0
	Figure 8K	2	0	0					
	Value of H	0.68547	0.83363	0.59182					

**TABLE 3:** Statistical numbers of the loops with various structures for the adding mechanism on different cellular spaces starting from the initial configuration in Figure 7A.

Loop Size	Shape∖Amount∖Space	60*60	80*65	100*65	Loop Size	Shape∖Amount∖Space	60*60	80*65	100*65
20 cells	Figure 8A	38	44	49	10 cells	Figure 8N	0	1	0
18 cells	Figure 8B	11	10	12		Figure 8O	0	0	2
	Figure 8C	0	0	1	8 cells	Figure 8P	12	18	19
16 cells	Figure 8F	1	1	1		Figure 8R	1	2	5
	Figure 8G	2	1	0	6 cells	Figure 8S	63	163	212
12 cells	Figure 8L	0	5	2	4 cells	Figure 8T	58	82	103
10 cells	Figure 8M	17	22	41					
	Value of H	0.72329	0.66301	0.65614					

**TABLE 4:** Statistical numbers of the loops with various structures for the changing mechanism on different cellular spaces starting from the initial configuration in Figure 7A.

Loop Size	Shape∖Amount∖Space	60*60	80*65	100*65	Loop Size	Shape∖Amount∖Space	60*60	80*65	100*65
20 cells	Figure 8A	32	35	36	12 cells	Figure 8K	0	1	0
18 cells	Figure 8B	0	0	1		Figure 8L	3	0	3
16 cells	Figure 8D	2	0	3	10 cells	Figure 8M	4	1	9
	Figure 8E	0	1	6		Figure 8N	2	2	4
	Figure 8F	0	1	0	8 cells	Figure 8P	32	6	54
	Figure 8G	11	12	15		Figure 8Q	11	12	20
14 cells	Figure 8I	2	3	5	6 cells	Figure 8S	29	115	137
12 cells	Figure 8J	23	14	22	4 cells	Figure 8T	34	139	77
	Value of H	0.91211	0.66984	0.85149					

**FIGURE 9:**
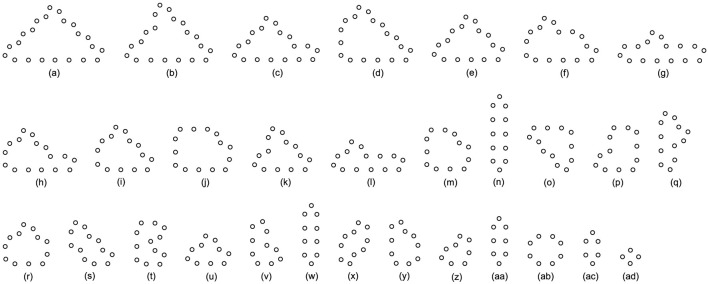
All final loop structures starting from the configuration in Figure 7B by the greedy selection, adding, and changing mechanisms.

**FIGURE 10:**
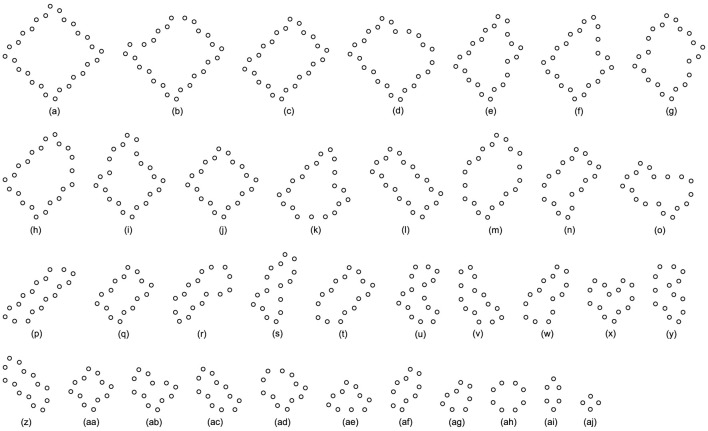
All final loop structures starting from the configuration in Figure 7C by the greedy selection, adding, and changing mechanisms.

Likewise, Tables 5, 6, and 7 provide the self-replication results starting from the initial configuration in Figure 7B, along with all possible final sub-loops given in Figure 9. The value of H of the greedy selection mechanism is lower than that of adding mechanism and changing mechanism, which means that the adding mechanism and the changing mechanism can give rise to more diversity. Moreover, small loops appear later in the changing mechanism than in the adding mechanism, leaving more room for larger loops to self-replicate and bring more kinds of species. In addition, Tables 8, 9, and 10 demonstrate the results from the initial configuration in Figure 7C by each mechanism, in which the greedy selection mechanism can achieve the highest value of H in 100*100 cellular space. All possible loop structures are shown in Figure 10. Though the kinds of loops are the least for greedy selection mechanism, there is the maximum number of loops. Therefore, in the same biological environment, when the kinds of species are relatively small and the population is relatively large, the species also have a high diversity. Especially, the adding mechanism can produce many loops with complete quantity and different sizes.

**TABLE 5:** Statistical numbers of the loops with various structures for the greedy selection mechanism on different cellular spaces starting from the initial configuration in Figure 7B.

Loop Size	Shape∖Amount∖Space	60*60	80*65	85*65	Loop Size	Shape∖Amount∖Space	60*60	80*65	85*65
22 cells	Figure 9A	66	36	60	8 cells	Figure 9Z	8	108	3
16 cells	Figure 9E	7	0	2	6 cells	Figure 9AC	0	0	5
10 cells	Figure 9U	8	0	47	4 cells	Figure 9AD	29	52	51
	Value of H	0.52218	0.43068	0.57119					

**TABLE 6:** Statistical numbers of the loops with various structures for the adding mechanism on different cellular spaces starting from the initial configuration in Figure 7B.

Loop Size	Shape∖Amount∖Space	60*60	80*65	85*65	Loop Size	Shape∖Amount∖Space	60*60	80*65	85*65
22 cells	Figure 9A	38	52	43	12 cells	Figure 9M	3	0	0
20 cells	Figure 9B	0	4	1		Figure 9N	35	0	1
18 cells	Figure 9C	0	1	1		Figure 9O	0	1	5
16 cells	Figure 9E	0	1	0		Figure 9P	0	0	3
	Figure 9F	0	1	0	10 cells	Figure 9V	1	0	26
	Figure 9G	0	0	1		Figure 9W	4	0	0
14 cells	Figure 9I	1	0	1	8 cells	Figure 9Z	1	16	2
	Figure 9J	2	1	0		Figure 9A	6	0	8
	Figure 9K	1	0	0	6 cells	Figure 9AC	53	14	18
	Figure 9L	0	1	0	4 cells	Figure 9AD	103	106	142
	Value of H	0.69327	0.57124	0.62362					

**TABLE 7:** Statistical numbers of the loops with various structures for the changing mechanism on different cellular spaces starting from the initial configuration in Figure 7B.

Loop Size	Shape∖Amount∖Space	60*60	80*65	85*65	Loop Size	Shape∖Amount∖Space	60*60	80*65	85*65
22 cells	Figure 9A	47	55	41	10 cells	Figure 9V	1	0	0
16 cells	Figure 9H	1	1	0		Figure 9X	1	4	56
12 cells	Figure 9N	1	0	1		Figure 9Y	3	28	0
	Figure 9Q	2	5	35	8 cells	Figure 9Z	4	7	1
	Figure 9R	31	0	0		Figure 9AA	1	0	0
	Figure 9S	0	1	1		Figure 9AB	18	2	0
	Figure 9T	0	0	1	6 cells	Figure 9AC	9	6	13
10 cells	Figure 9U	1	0	1	4 cells	Figure 9AD	16	43	19
	Value of H	0.81213	0.71099	0.70811					

**TABLE 8:** Statistical numbers of the loops with various structures for the greedy selection mechanism on different cellular spaces starting from the initial configuration in Figure 7C.

Loop Size	Shape∖Amount∖Space	60*60	80*80	100*100	Loop Size	Shape∖Amount∖Space	60*60	80*80	100*100
28 cells	Figure 10A	36	83	57	12 cells	Figure 10AA	59	2	104
24 cells	Figure 10C	0	6	25	8 cells	Figure 10AG	7	2	83
20 cells	Figure 10J	0	4	9	6 cells	Figure 10AI	0	0	5
16 cells	Figure 10Q	14	25	71	4 cells	Figure 10AJ	1	63	47
	Value of H	0.50862	0.55976	0.79217					

**TABLE 9:** Statistical numbers of the loops with various structures for the adding mechanism on different cellular spaces starting from the initial configuration in Figure 7C.

Loop Size	Shape∖Amount∖Space	60*60	80*80	100*100	Loop Size	Shape∖Amount∖Space	60*60	80*80	100*100
28 cells	Figure 10A	42	86	141	16 cells	Figure 10Q	2	0	0
26 cells	Figure 10B	0	1	4		Figure 10R	1	0	0
24 cells	Figure 10C	0	0	20		Figure 10S	1	0	0
	Figure 10D	14	0	0		Figure 10T	0	0	1
22 cells	Figure 10E	4	0	0	14 cells	Figure 10V	1	0	0
	Figure 10F	0	4	0	12 cells	Figure 10AA	0	2	0
	Figure 10G	0	0	2		Figure 10AB	1	1	0
	Figure 10H	0	0	3	10 cells	Figure 10AE	0	3	0
22 cells	Figure 10I	0	0	1		Figure 10AF	0	0	2
20 cells	Figure 10J	0	25	0	8 cells	Figure 10AG	5	1	1
	Figure 10K	0	1	0	6 cells	Figure 10AI	0	1	1
18 cells	Figure 10N	1	0	0	4 cells	Figure 10AJ	16	8	83
	Figure 10O	1	0	0					
	Value of H	0.71351	0.52249	0.50833					

**TABLE 10:** Statistical numbers of the loops with various structures for the changing mechanism on different cellular spaces starting from the initial configuration in Figure 7C.

Loop Size	Shape∖Amount∖Space	60*60	80*80	100*100	Loop Size	Shape∖Amount∖Space	60*60	80*80	100*100
28 cells	Figure 10A	43	72	138	12 cells	Figure 10AA	0	1	2
20 cells	Figure 10J	2	2	5		Figure 10AC	0	1	1
	Figure 10I	0	15	19		Figure 10AD	0	0	4
	Figure 10M	0	0	1	10 cells	Figure 10AF	1	69	0
18 cells	Figure 10P	19	3	0	8 cells	Figure 10AG	1	6	0
16 cells	Figure 10U	0	7	0		Figure 10AH	0	0	1
14 cells	Figure 10X	5	4	29	6 cells	Figure 10AI	3	1	26
	Figure 10Y	0	1	0	4 cells	Figure 10AJ	1	44	2
	Figure 10Z	0	1	0					
	Value of H	0.54088	0.74551	0.57765					

**FIGURE 11:**
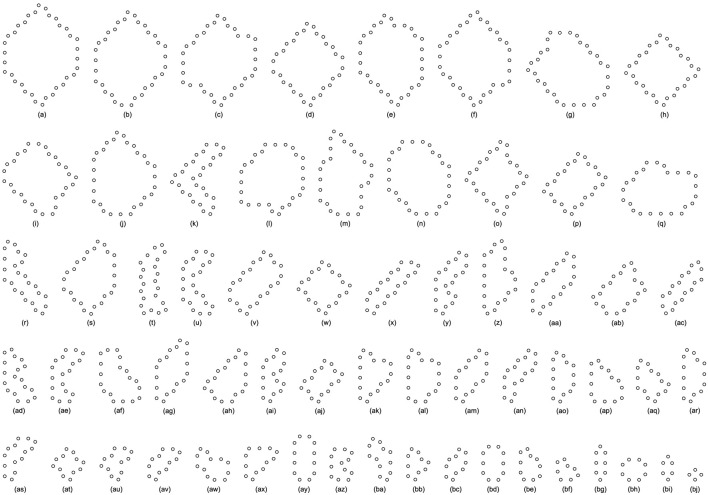
All final loop structures starting from the configuration in Figure 7D by the greedy selection, adding, and changing mechanisms.

**FIGURE 12:**
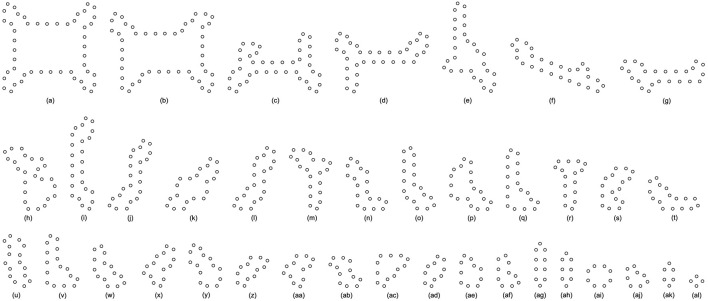
All final loop structures starting from the configuration in Figure 7E by the greedy selection, adding, and changing mechanisms.

All replicating results of the loop structures from the configuration in Figure 7D are given in Figure 11. In this case, the values of H using the adding mechanism and the changing mechanism in Tables 12, 13, respectively are obviously higher than that of the greedy selection mechanism in Table 11. Furthermore, self-replications starting from the irregular and symmetric shapes in Figure 7E are elaborated in Tables 14, 15, and 16 with various types of sub-loops shown in Figure 12. It can be verified that the loop that is the same as the initial configuration quickly takes up the entire space, leaving little room for the smaller ones, which creates a smaller population of loops and owns the lowest diversity of species.

**TABLE 12:** Statistical numbers of the loops with various structures for the adding mechanism on different cellular spaces starting from the initial configuration in Figure 7D.

Loop Size	Shape∖Amount∖Space	60*60	80*80	100*100	Loop Size	Shape∖Amount∖Space	60*60	80*80	100*100
34 cells	Figure 11A	32	47	86	14 cells	Figure 11AO	0	1	0
32 cells	Figure 11B	2	1	0		Figure 11AP	0	1	0
	Figure 11C	0	0	1		Figure 11AQ	0	0	7
30 cells	Figure 11E	1	0	1		Figure 11AR	0	0	1
	Figure 11F	0	0	2	12 cells	Figure 11AT	0	4	0
	Figure 11G	0	0	1		Figure 11AV	1	0	1
28 cells	Figure 11J	4	0	2		Figure 11AW	1	0	0
	Figure 11K	0	1	0		Figure 11AX	0	20	0
26 cells	Figure 11L	0	0	1		Figure 11AY	0	1	0
	Figure 11M	0	0	1		Figure 11AZ	0	2	0
24 cells	Figure 11Q	1	0	0		Figure 11AB	0	0	1
	Figure 11R	0	1	1	10 cells	Figure 11BC	0	1	0
20 cells	Figure 11Y	0	1	0		Figure 11BD	1	0	0
	Figure 11Z	0	0	1		Figure 11BE	0	3	0
	Figure 11AA	0	0	3	8 cells	Figure 11BF	5	3	17
18 cells	Figure 11AC	1	0	0		Figure 11BG	3	0	1
	Figure 11AD	0	0	4		Figure 11BH	0	1	0
16 cells	Figure 11AK	0	9	0	6 cells	Figure 11BI	5	51	29
	Figure 11AL	0	1	0	4 cells	Figure 11BJ	4	33	30
	Figure 11AM	0	0	1					
	Value of H	0.76886	0.85201	0.79910					

**TABLE 13:** Statistical numbers of the loops with various structures for the changing mechanism on different cellular spaces starting from the initial configuration in Figure 7D.

Loop Size	Shape∖Amount∖Space	60*60	80*80	100*100	Loop Size	Shape∖Amount∖Space	60*60	80*80	100*100
34 cells	Figure 11A	26	40	64	16 cells	Figure 11AN	0	0	1
26 cells	Figure 11N	0	0	1	14 cells	Figure 11AS	0	3	0
	Figure 11O	0	0	3	12 cells	Figure 11AU	13	39	2
24 cells	Figure 11S	0	0	2		Figure 11AV	0	0	3
22 cells	Figure 11T	2	22	1	10 cells	Figure 11BB	31	13	77
	Figure 11U	2	0	0		Figure 11BC	10	0	0
	Figure 11V	0	0	1		Figure 11BE	0	1	0
20 cells	Figure 11AB	1	0	0	8 cells	Figure 11BF	0	20	2
18 cells	Figure 11AE	1	0	0		Figure 11BG	0	35	2
	Figure 11AF	0	2	0	6 cells	Figure 11BI	6	5	32
	Figure 11AG	0	0	1	4 cells	Figure 11BJ	3	14	187
	Figure 11AH	0	0	1					
	Value of H	0.76923	0.88685	0.63472					

**TABLE 11:** Statistical numbers of the loops with various structures for the greedy selection mechanism on different cellular spaces starting from the initial configuration in Figure 7D.

Loop Size	Shape∖Amount∖Space	60*60	80*80	100*100	Loop Size	Shape∖Amount∖Space	60*60	80*80	100*100
34 cells	Figure 11A	30	34	43	12 cells	Figure 11AT	19	0	0
30 cells	Figure 11D	0	0	1		Figure 11AU	0	123	0
28 cells	Figure 11H	5	0	9	10 cells	Figure 11BB	4	0	0
24 cells	Figure 11P	0	0	1		Figure 11BC	0	0	249
20 cells	Figure 11W	0	2	0	8 cells	Figure 11BF	0	34	20
	Figure 11X	0	0	1		Figure 11BG	0	0	1
16 cells	Figure 11AI	0	1	22	6 cells	Figure 11BI	0	2	2
	Figure 11AJ	0	0	1	4 cells	Figure 11BJ	12	31	9
	Value of H	0.59562	0.55587	0.49319					

**TABLE 14:** Statistical numbers of the loops with various structures for the greedy selection mechanism on different cellular spaces starting from the initial configuration in Figure 7E.

Loop Size	Shape∖Amount∖Space	60*46	80*65	85*65	Loop Size	Shape∖Amount∖Space	60*46	80*65	85*65
48 cells	Figure 12A	19	27	22	14 cells	Figure 12W	0	0	4
46 cells	Figure 12B	1	0	0	10 cells	Figure 12AD	9	10	4
28 cells	Figure 12E	1	0	0	8 cells	Figure 12AH	2	4	0
22 cells	Figure 12J	0	0	6	6 cells	Figure 12AK	0	0	25
16 cells	Figure 12R	0	4	0	4 cells	Figure 12AL	1	8	0
	Figure 12S	0	0	1					
	Value of H	0.50377	0.57922	0.59937					

**TABLE 15:** Statistical numbers of the loops with various structures for the adding mechanism on different cellular spaces starting from the initial configuration in Figure 7E.

Loop Size	Shape∖Amount∖Space	60*46	80*65	85*65	Loop Size	Shape∖Amount∖Space	60*46	80*65	85*65
48 cells	Figure 12A	12	15	18	14 cells	Figure 12X	3	1	8
40 cells	Figure 12C	2	0	0	12 cells	Figure 12Z	0	2	3
34 cells	Figure 12D	0	2	2		Figure 12AA	0	2	1
24 cells	Figure 12G	1	0	0	10 cells	Figure 12AD	3	4	34
	Figure 12H	0	0	2		Figure 12AE	0	2	2
	Figure 12I	0	0	1	8 cells	Figure 12AH	0	0	1
20 cells	Figure 12K	1	0	0		Figure 12AI	3	1	0
	Figure 12L	0	0	11		Figure 12AJ	69	108	12
18 cells	Figure 12N	2	0	0	6 cells	Figure 12AK	5	18	6
	Figure 12O	0	1	0	4 cells	Figure 12AL	12	21	53
16 cells	Figure 12T	0	1	0					
	Value of H	0.62145	0.60756	0.85253					

**TABLE 16:** Statistical numbers of the loops with various structures for the changing mechanism on different cellular spaces starting from the initial configuration in Figure 7E.

Loop Size	Shape∖Amount∖Space	60*46	80*65	85*65	Loop Size	Shape∖Amount∖Space	60*46	80*65	85*65
48 cells	Figure 12A	11	16	12	12 cells	Figure 12AB	2	0	0
26 cells	Figure 12F	0	0	1		Figure 12AC	0	1	0
24 cells	Figure 12G	0	0	1	10 cells	Figure 12AE	1	0	1
20 cells	Figure 12M	0	1	0		Figure 12AF	11	47	50
18 cells	Figure 12P	5	0	0		Figure 12AG	0	1	0
	Figure 12Q	0	4	0	8 cells	Figure 12AI	0	1	0
16 cells	Figure 12U	24	0	1		Figure 12AJ	5	0	60
	Figure 12V	0	1	0	6 cells	Figure 12AK	11	44	4
14 cells	Figure 12X	4	6	10	4 cells	Figure 12AL	14	11	48
	Figure 12Y	0	0	2					
	Value of H	0.88156	0.70506	0.70877					

Therefore, the aforementioned experiments show that the adding mechanism and the changing mechanism can bring higher diversity than the greedy selection mechanism. Moreover, for those loops with the same number of cells, the adding mechanism and the changing mechanism can obtain more variable loops with different phenotypes. Phenotype change is a sufficient factor for achieving such a functional evolution Kampis and Gulyás (2008). In the process of self-replicating, once a minimal loop is created, the loop will quickly replicate itself, because the minimal loop can track its body much faster. As a result, the minimal loops will become the vast majority of the population after reaching saturation, thereby reducing the diversity. Such a tendency is similar to the basic orientation of the evolution paths in Sayama (2004).

**FIGURE 13:**
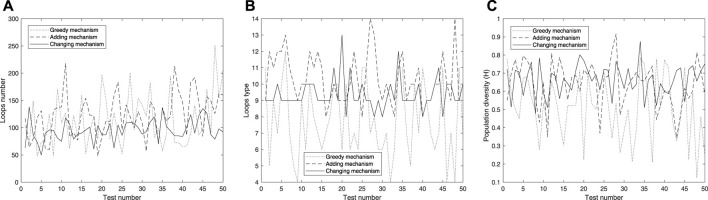
Further results on the initial configuration in Figure 7D with 60*60 cellular space using the three mechanisms.

Moreover, in order to further test the diversity that the active mechanisms can bring, we conducted experiments on the initial configuration in 7(d) with 60*60 cellular space using three mechanisms. From Figure 13, we can see that the greedy mechanism mostly can obtain the highest value on the total quantity of loops, but significantly lower than the active mechanisms in terms of species and diversity, which may imply that the greedy mechanism tends to produce smaller loops. Generally speaking, smaller loops can replicate themselves rapidly and be more likely to survive.

**FIGURE 14:**
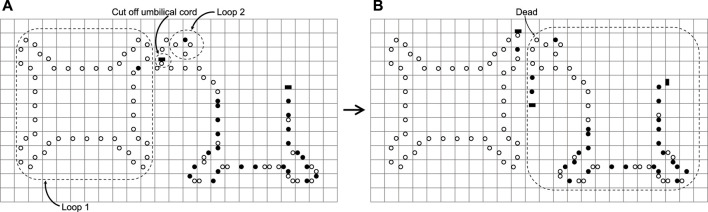
A dead loop caused by improperly cutting off an umbilical cord.

However, mistakes may occur in the process of self-replication and the details are shown in Figure 14. There are several conditions for the error to occur (see also Huang et al. (2013)): 1) Loop 1 is on the inner side of the arm of the loop 2 in Figure 14A; 2) The arm of loop 1 contains no construction code, which means the head of the arm is in the state ◦■; 3)The construction arm of loop 2 has been scanned by a validation signal, which means the state about the part of the arm turns state ***•*** to state ◦. Especially, there is a parallel arm that is made up of state ◦ shown in Figure 14B. However, this error seldom happens. Under these conditions, loop 2 may have an erroneous cognition that it thinks of the arm of loop 2 as its own; thereby it will cut off the umbilical cord at the arm head. Fortunately, loop 1 is unaffected by this error and goes on self-replicating. Loop 2, however, is not so lucky, and dies. What is worse, the dead loop 2 and the discarded arm of loop 1 waste many spaces. Nevertheless, enhancing the function of a validation signal may seem reasonable to avoid erroneous cognition. On the plus side, an erroneous cognition may possibly be regarded as some non-trivial co-action between loops Sayama (1999). Moreover, an erroneous cognition may create an offspring the size of which is bigger than the mother loop Salzberg (2003).

**FIGURE 15:**
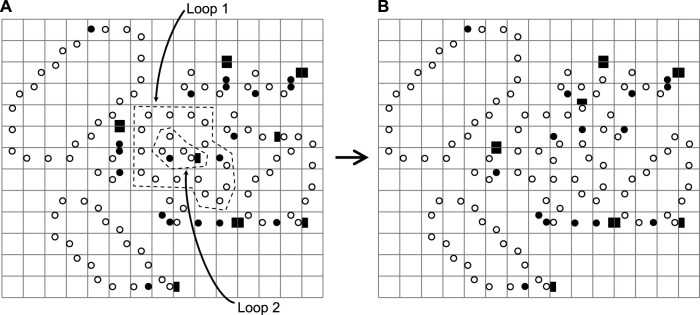
A phagocytosis situation because of different self-replicating speeds.

Furthermore, from Figure 15, we can see that Loop 2 takes up the space thanks to the faster replication capability during the process of generating Loop 1, and Loop 1 exactly forms a closed loop that wraps around Loop 2. This situation is similar to the phagocytosis of immune cell Stossel (1974). Luckily, Loop 1 and Loop 2 are alive. Thus, if there are enough spaces, the loops can self-replicate.

## 5 Discussion

Many studies have considered the self-replication on various cellular automata to simulate the process of biological self-replication, including the reversible cellular automata (Morita and Imai, 1996b), polymorphic cellular automata (Sekanina and Komenda, 2011), and graph automata (Tomita et al., 2002). Moreover, self-replication on cellular automata has been applied to several fields, such as worm propagation in smartphones (Peng et al., 2013), artificial chemistry (Hutton, 2007), and image processing (Sahin et al., 2015). In this article, we provided a different approach to enhance the diversity of artificial self-replicating structures, instead of abandoning partial structural information or destroying the whole loop. In order to obtain these effects better, on the basis of existing ordinary self-replication, we change a greedy selection mechanism to two active mechanisms when dealing with collision, which add an orientation and change the construction signal under the dead head. Experiments showed that active adaptations using our schemes can actually improve the possibility of survival and replication of any self-replicating structure in a wide variety of environments than the passive one. In particular, the changing mechanism involves abandoning one building-block from the original structure of a mother loop when every collision happens, even though the mechanism changes the construction signal. Also, the adding mechanism does not seem to lose the block of information coming from the parent, while some constructional information is left for the offspring to complete the replication. This may result in the shrinkage of both shape and size of the offspring.

Although the adding and changing mechanisms enable more active self-adaptation than the greedy selection mechanism, they still look somewhat passive in the sense that the adaptation can only be activated when collision occurs. In living organisms, mutation on genes will occur in a probabilistic manner. As with self-adaptation, self-recovery or self-healing is also an interesting feature of organisms. In the future work, we will consider how to endow self-replicating loops with a self-repairing ability (Tempesti et al., 1998), use random inputs (Griffith et al., 2005) to generate interesting patterns, and genetic algorithms to automatically discover rules (Lohn and Reggia, 1997).

## Data Availability

The original contributions presented in the study are included in the article/Supplementary Material; further inquiries can be directed to the corresponding author.
