# Supplementary material for: Enhancing the diversity of self-replicating structures using active self-adapting mechanisms
Source: Front Genet. 2022 Jul 26;13:958069. doi: 10.3389/fgene.2022.958069 (PMC9360575; doi:10.3389/fgene.2022.958069)

# Supplementary Material

## 1 APPENDIX A

Transition rules $f(s_1, s_2, s_3, s_4, s_5, s_6, s_7, s_8) = (s_9, s_{10}, s_{11}, s_{12}, s_{13}, s_{14}, s_{15}, s_{16})$ with $\forall i \in \{1, ..., 16\} \exists j \in \{1, ..., 8\}$ $(s_i \in \{\sharp, \circ, \bullet, \blacksquare\} \wedge s_j \neq s_{j+8})$ are listed below, with their rotational symmetry equivalents left out.

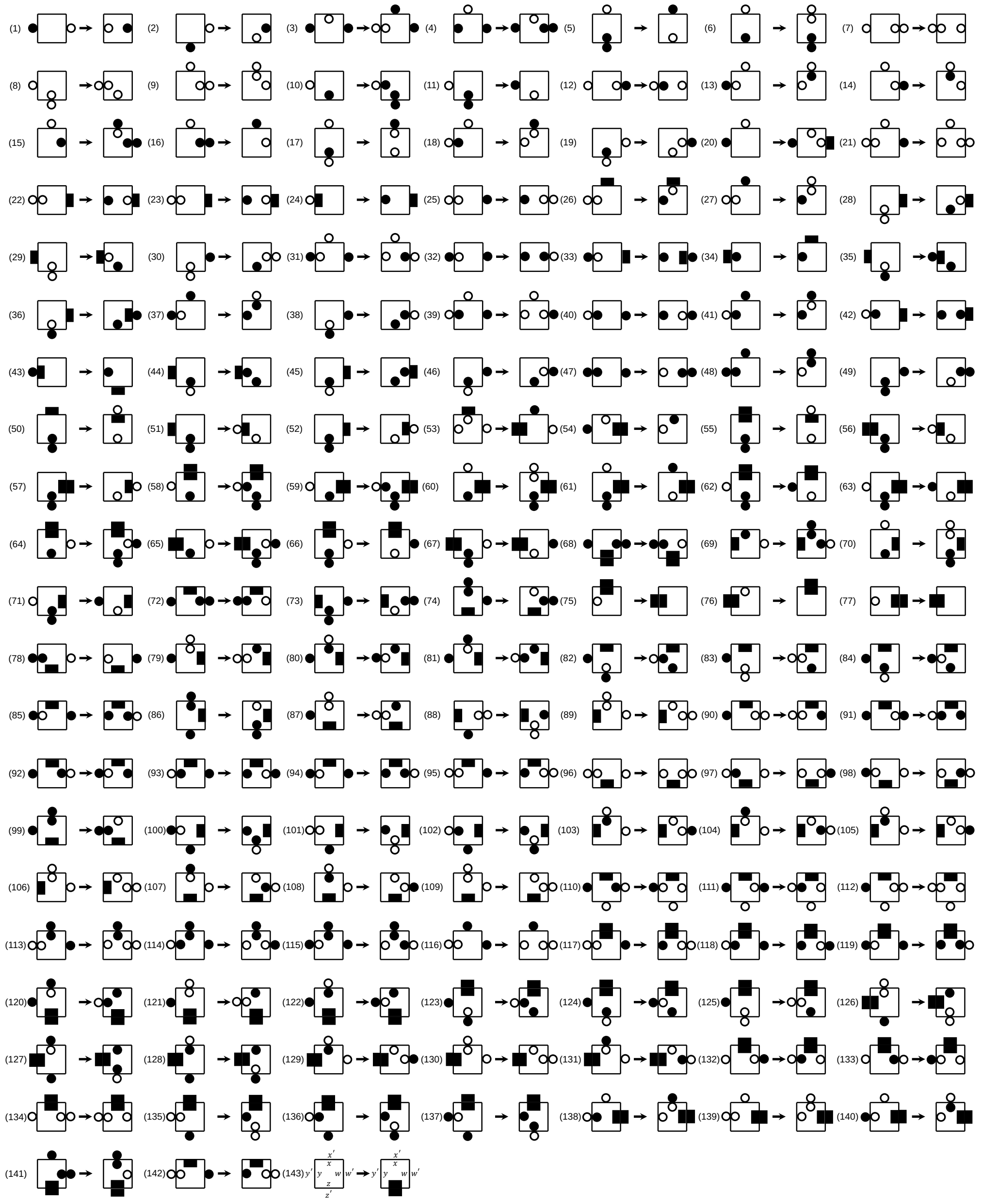

The following rules are used for adding mechanism.

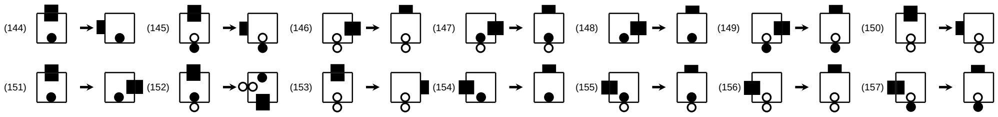


Below are rules for changing mechanism.

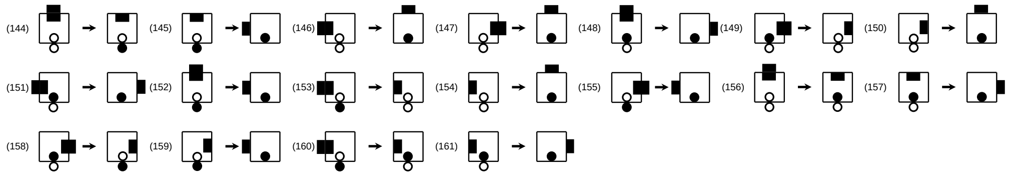


where $(x, x'), (y, y'), (w, w') \in \{\sharp, \circ, \bullet, \blacksquare\}^2$ and $(z, z') \in \{\blacksquare\} \times \{\sharp, \circ, \bullet\} \cup \{\sharp, \circ, \bullet\} \times \{\blacksquare\}$. In particular, the rule 143 employs wild cards for cell states in both the left-hand and right-hand sides, which actually stands for a set of rules obtained by substituting each wild card with a certain state. From this set, however,we must exclude every rule (as well as its rotational symmetry equivalents) of which the left-hand side $(x, y, z, w, x', y', z', w')$ coincides with one of the following state patterns.

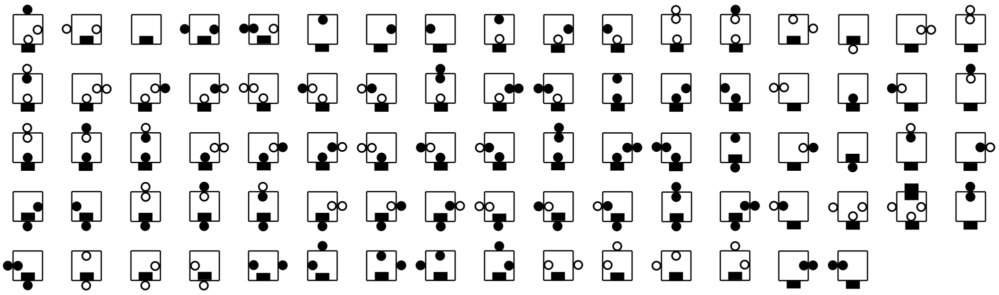

Supplement: Supplementary file 1 [file DataSheet1.pdf]
